# Evaluating Uncertainty to Strengthen Epidemiologic Data for Use in Human Health Risk Assessments

**DOI:** 10.1289/ehp.1308062

**Published:** 2014-07-31

**Authors:** Carol J. Burns, J. Michael Wright, Jennifer B. Pierson, Thomas F. Bateson, Igor Burstyn, Daniel A. Goldstein, James E. Klaunig, Thomas J. Luben, Gary Mihlan, Leonard Ritter, A. Robert Schnatter, J. Morel Symons, Kun Don Yi

**Affiliations:** 1 The Dow Chemical Company, Midland, Michigan, USA; 2 National Center for Environmental Assessment (NCEA), U.S. Environmental Protection Agency (EPA), Cincinnati, Ohio, USA; 3 Health and Environmental Sciences Institute, International Life Sciences Institute (ILSI-HESI), Washington, DC, USA; 4 NCEA, U.S. EPA, Washington, DC, USA; 5 Department of Environmental and Occupational Health, Drexel University School of Public Health, Philadelphia, Pennsylvania, USA; 6 Monsanto, St. Louis, Missouri, USA; 7 Department of Environmental Health, Indiana University School of Public Health, Bloomington, Indiana, USA; 8 NCEA, U.S. EPA, Research Triangle Park, North Carolina, USA; 9 Bayer CropScience, Research Triangle Park, North Carolina, USA; 10 School of Environmental Sciences, University of Guelph, Guelph, Ontario, Canada; 11 ExxonMobil Biomedical Sciences Inc., Annandale, New Jersey, USA; 12 E.I. du Pont de Nemours and Company, Newark, Delaware, USA; 13 Syngenta Crop Protection LLC, Greensboro, North Carolina, USA

## Abstract

Background: There is a recognized need to improve the application of epidemiologic data in human health risk assessment especially for understanding and characterizing risks from environmental and occupational exposures. Although there is uncertainty associated with the results of most epidemiologic studies, techniques exist to characterize uncertainty that can be applied to improve weight-of-evidence evaluations and risk characterization efforts.

Methods: This report derives from a Health and Environmental Sciences Institute (HESI) workshop held in Research Triangle Park, North Carolina, to discuss the utility of using epidemiologic data in risk assessments, including the use of advanced analytic methods to address sources of uncertainty. Epidemiologists, toxicologists, and risk assessors from academia, government, and industry convened to discuss uncertainty, exposure assessment, and application of analytic methods to address these challenges.

Synthesis: Several recommendations emerged to help improve the utility of epidemiologic data in risk assessment. For example, improved characterization of uncertainty is needed to allow risk assessors to quantitatively assess potential sources of bias. Data are needed to facilitate this quantitative analysis, and interdisciplinary approaches will help ensure that sufficient information is collected for a thorough uncertainty evaluation. Advanced analytic methods and tools such as directed acyclic graphs (DAGs) and Bayesian statistical techniques can provide important insights and support interpretation of epidemiologic data.

Conclusions: The discussions and recommendations from this workshop demonstrate that there are practical steps that the scientific community can adopt to strengthen epidemiologic data for decision making.

Citation: Burns CJ, Wright JM, Pierson JB, Bateson TF, Burstyn I, Goldstein DA, Klaunig JE, Luben TJ, Mihlan G, Ritter L, Schnatter AR, Symons JM, Yi KD. 2014. Evaluating uncertainty to strengthen epidemiologic data for use in human health risk assessments. Environ Health Perspect 122:1160–1165; http://dx.doi.org/10.1289/ehp.1308062

## Introduction

Human health risk assessments have traditionally relied heavily on toxicologic and other experimental data, but there is an increased recognition of the value of using epidemiologic data in risk assessment. Previous publications (Fann et al. 2011; Jones et al. 2009; Lavelle et al. 2012; Vlaanderen et al. 2008) and initiatives have discussed how to improve the application of these epidemiologic data to risk assessments. As an example, at a meeting held in early 2010, the U.S. Environmental Protection Agency (EPA) requested input from the Federal Insecticide, Fungicide and Rodenticide Act Scientific Advisory Panel (FIFRA SAP) on approaches for the “[i]ncorporation of epidemiology and human incident data into human health risk assessment[s]” (U.S. EPA 2009a). Epidemiologic studies play a key role in setting national ambient air quality standards (U.S. EPA 2009b) and contribute substantially to other thematic weight-of-evidence approaches toward evaluating causality based on multiple lines of evidence (Rhomberg et al. 2010; Weed 2005).

The incorporation of epidemiologic evidence into risk assessments is an important part of understanding and characterizing risks from environmental and occupational exposures. Uncertainty arises from study limitations regarding internal validity including exposure assessment, confounding and other potential sources of bias, and external validity or generalization from study populations to the populations for which risk assessments are conducted (Guzelian et al. 2005; Hertz-Picciotto 1995; Lash et al. 2009; Levy 2008; Maldonado 2008; Persad and Cooper 2008). Further, point estimates can be inaccurate because of internal validity issues and also because confidence intervals focus only on the potential for random error. These different sources of uncertainty can have an impact on various steps of the risk assessment paradigm (including hazard identification, exposure assessment, and dose–response assessment) resulting in hazards that are not recognized, hazards that are incorrectly identified, or inaccurate dose–response characterizations that may lead to over- or underestimation of “safe” exposure levels.

Epidemiologic approaches and statistical techniques exist to characterize uncertainty that can be applied to weight-of-evidence evaluations and risk characterization efforts. Although there is strong theoretical support for the utility of these approaches, their translation into regular epidemiologic practice is lagging. In addition, the impact of potential sources of error in epidemiologic studies is often only qualitatively discussed. For example, with respect to exposure measurement error, Jurek et al. (2006) sampled papers from three epidemiology journals over 1 year and found that only 61% of the articles made any mention of exposure measurement error, and only 46% of those qualitatively described the possible effects. Only 1 of 57 sampled studies quantified the likely impact of exposure measurement error on results. This incomplete information demonstrates an opportunity among epidemiologists to characterize the magnitude and impact of various sources of uncertainty, which can help address one of the more difficult challenges in risk assessment.

This report derives from a workshop held in Research Triangle Park, North Carolina, in October 2012 (http://www.hesiglobal.org/i4a/pages/index.cfm?pageID=3641) to discuss the utility of using epidemiologic data in risk assessments, including the use of advanced analytic methods to address sources of uncertainty. The objective of the workshop was to develop recommendations on strengthening epidemiologic studies so that these data can more effectively be integrated in risk assessments. The Health and Environmental Sciences Institute (HESI) workshop was focused specifically on uncertainty, exposure assessment, and application of analytic methods to address these challenges. Cross-disciplinary experts in epidemiology, toxicology, exposure assessment, and risk assessment attended the workshop. The deliberations highlighted opportunities for epidemiologists to enhance scientific research in general and to address issues related to the development and use of epidemiologic data in risk assessment.

## Uncertainty

The National Research Council (NRC 2009) defined uncertainty as the “lack or incompleteness of information” critical for the risk assessment process. Uncertainty in an epidemiologic study can arise from both random and systematic error in the study, whereas uncertainty in a risk assessment can arise from internal and external validity concerns arising from one study or set of studies included in the assessment. Thus, the characterization of scientific uncertainty can provide risk assessments with a level of confidence regarding decisions that are being made and allows for evaluation of the degree that uncertainty plays in the analysis of consequences of specific policies. The NRC (2009) recommended that “risk assessments should characterize and communicate uncertainty and variability in all key computational steps of risk assessments” while recognizing that “uncertainty analysis and characterization pose difficult technical issues, and in general related best practices have not been established.” Thus, determining the nature and magnitude of uncertainties remains one of the key challenges in risk assessment.

Because results across epidemiologic, toxicologic, and clinical data may be discordant at times, there is a distinct need to understand and characterize sources of uncertainty within each of these areas to characterize potential risk and hazard for risk assessment purposes. A comprehensive analysis of uncertainty across all data sources can act as a bridge to foster the integration necessary to focus further research, improve risk assessment, and understand potential impacts on human health.

### Uncertainty Issues and Recommendations

*Improved characterization and discussion of plausible sources of uncertainty would be beneficial in all epidemiologic reports and publications.* The potential for bias in epidemiologic studies is routinely acknowledged in published reports but is nearly always limited to a qualitative discussion (Jurek et al. 2006). Even quantitative discussions of, for example, selection bias are typically limited to examinations of participation rates or to potential sampling bias due to self-selection. In addition, the potential for residual confounding by measured or unmeasured factors is often acknowledged, but the magnitude and direction are usually unknown or unstated. Thus, characterizing and documenting the relationships (i.e., the direction and magnitude of associations) among potential confounders, exposures, and outcomes of interest is critical. Knowing the direction of a potential confounder (e.g., positive vs. negative confounding) could enable epidemiologic data to be used in the hazard identification stage of a risk assessment or for dose–response assessments if the magnitude of confounding is also known or the uncertainty from this source of bias could be quantified. Addressing these possible sources of bias in an epidemiologic study may allow risk assessors, to the extent possible, to quantify the consequences of any bias in a specific study or across a group of similar studies. Although not a type of bias, an additional source of uncertainty is related to generalizing study results beyond the sample population being examined in an epidemiologic study. Characterizing variability in risk among different susceptible populations will ultimately make results of epidemiologic studies more relevant to risk assessment efforts and risk management decision making.

*Conduct more validation studies and uncertainty analyses of epidemiologic study findings.* The overall impact of different sources of uncertainty on epidemiologic results is infrequently considered in epidemiologic publications, and data sufficient to allow the reader to undertake independent uncertainty assessments are often not presented (Jurek et al. 2007). This is essentially the lowest tier (i.e., tier 0) of uncertainty analyses recognized by the NRC (2009):

Tier 0: Default assumptions—single value of result
Tier 1: Qualitative but systematic identification and characterization of uncertainty
Tier 2: Quantitative evaluation of uncertainty making use of bounding values, interval analysis, and sensitivity analysis
Tier 3: Probabilistic assessment with single or multiple outcome distributions reflecting uncertainty and variability.

We therefore recommend that investigators obtain additional data needed to facilitate uncertainty analysis and undertake at least a qualitative assessment of uncertainty, including all recognized sources or justifying their omission. A qualitative characterization would include identifying possible sources and beginning to assess the sources, direction, and magnitude of uncertainty. The potential ramifications of each source of uncertainty should be addressed and some crude classification or categorization approaches could be developed (e.g., low, intermediate, or high uncertainty) with respect to a given source. When sources of uncertainty can be identified but not fully quantified within a study or set of studies, there may be default data available that can be useful in estimating a possible range of values (Stürmer et al. 2007). Indeed, there may also be a complete lack of data that contribute to the uncertainty. However, investigators could consider the direction and magnitude of the potential uncertainty (i.e., confounding and/or bias). Such data would allow for higher tiers of uncertainty analyses. Methodologic guidance and software for quantitative bias analysis have also become available (Lash et al. 2009) but are not yet common in risk assessment. Ideally, to facilitate the highest tier of uncertainty analysis, a quantitative assessment of individual and conjoint sources of uncertainty would be included in every epidemiologic study. The conduct of more validation studies and sensitivity analyses is also recommended to better understand methodological issues and sources of uncertainty (Chatterjee and Wacholder 2002; Greenland 1996; Rosenbaum 2005; Schneeweiss 2006; VanderWeele and Arah 2011).

*Improve communication about epidemiologic uncertainty.* We encourage full disclosure of uncertainty in epidemiology as a matter of transparency. Characterization and quantification of uncertainty should increase such that the basis of decisions and assumptions are clear, either within the publication or in supplemental information. Epidemiologists and their peer scientists should encourage publications and other communications to include the necessary study-specific data on internal data relationships relevant to selection bias, information bias, and confounding for quantitative bias analysis to assess uncertainty (Lash et al. 2009). Reviewers of manuscripts should also recommend qualitative, and if possible quantitative, discussions of uncertainties. The objective is for such information to be more routinely collected and reported.

*Develop a broader matrix of sources of uncertainty for the overall risk assessment process, with the goal of harmonization of uncertainty assessment across different disciplines.* Risk assessments consider the totality of the evidence (i.e., epidemiology, toxicology, and other lines of scientific evidence, as well as any other knowledge in the context of risk) when determining the weight of evidence. Other lines of evidence such as toxicology and mode of action can be used to inform the interpretation and use of epidemiologic data in risk assessment. Because uncertainties exist in all lines of scientific endeavor, each source of uncertainty across these areas should be considered in assessing uncertainty in the overall risk assessment. Previous efforts have recommended harmonization of the incorporation of uncertainty in risk assessment, primarily focusing on the use of default uncertainty values from toxicologic data (Sonich-Mullin et al. 2001). Consequently, harmonization of sources of uncertainty across epidemiology and toxicology should be undertaken in a systematic manner that will make for more transparent decision making.

## Exposure Assessment

Exposure science aims to quantify the intensity, frequency, duration, and timing of human contact with chemical, physical, or biological agents occurring in the environment, and may be used to further inform evaluation of causality in the environmental source-to-health outcome continuum (Barr 2006). Within exposure science, exposure assessment specifically deals with several distinct aspects that underlie the risk assessment process, including exposure source(s), environmental pathway(s), environmental concentrations, human exposures, and dose.

Data are rarely available on biologically relevant dose metrics (e.g., absorbed dose, effective dose) in the organ or tissue of interest in epidemiologic studies; thus, dose is often estimated indirectly using exposure metrics. These surrogate estimates of exposure are subject to measurement error because they may rely on imperfectly measured concentrations in the individual, or on models of transport and fate in the environment or workplace. In addition, measurement error may result from estimates of the distribution of human uptake over time (e.g., use of a physiologically based pharmacokinetic model) or collection of activity pattern data.

Similar to measurement error and the resulting misclassification of health outcome data, exposure misclassification is important to characterize in epidemiologic studies because it can distort exposure–response relationships and lead to biased or imprecise results. Exposure measurement error can be differential or nondifferential with respect to variation in disease status. Exposure measurement error can lead to exposure misclassification when exposure surrogates for individual participants are classified into categories for analysis.

Differential misclassification can arise in categorical exposure metrics even when there is nondifferential error (i.e., independent of disease status) in an exposure variable that is measured on a continuous scale (Flegal et al. 1991). For epidemiologic studies to be evaluated and used appropriately in risk assessment, it is important that exposure measurement error is characterized and evaluated thoroughly with consideration of the magnitude and direction of any potential exposure misclassification bias (Bergen et al. 2013). This information is useful for risk assessors when they evaluate the potential for bias and confidence placed on study results.

### Exposure Assessment Issues and Recommendations

*An interdisciplinary perspective is needed during the study-design phase to ensure that biologically relevant quantitative exposure–response information is collected that will be useful for risk assessment purposes.* During the study-design phase, an interdisciplinary team including experts—for example, in epidemiology, exposure assessment, industrial hygiene, and analytical chemistry—should be assembled to develop robust exposure assessment approaches. This might include consideration of targeted data collection strategies, such as collection of exposure or surrogate data based on the appropriate biological matrix, sample number, and the critical exposure window(s). Other constraints that can be addressed include sources of exposure variability, availability of resources, participant burden, and ethical considerations (with institutional review board review as appropriate). This interdisciplinary approach will allow for the collection of biologically relevant exposure data to increase the potential for quantification of exposure–response relationships that will be useful for risk assessment and risk management purposes.

*Develop exposure assessment approaches that are transparent and well characterized.* We recommend that study authors should discuss the nature (i.e., type, direction, and magnitude) and likelihood of any expected exposure measurement error and misclassification bias. An evaluation of measurement error and any resulting impact on effect estimates would provide risk assessors with information to weight studies by the quality of the exposure assessment, the methods used to adjust for exposure measurement error, and the likelihood that exposure measurement error contributes to uncertainty in effect estimates in epidemiologic studies. Characterization of exposure data quality may include steps to make exposure data publicly available so that risk assessors can perform secondary data analyses including sensitivity and uncertainty analyses.

*Quantify exposure measurement error and examine and correct for its impact on effect estimates.* Ignoring uncertainty in exposure estimation can produce bias when such estimates are used to examine associations with adverse health effects (Carroll et al. 1995). Although epidemiologic publications infrequently present detailed information on the potential impact of measurement error (Jurek et al. 2006; Spiegelman 2010), epidemiologic study results would be enhanced by detailing exposure assessment assumptions and characterizing measurement error to allow risk assessors to gauge the potential impact of this error. This information should include characterization of different sources and types of measurement error. The sources and types can be based on various assumptions used in exposure modeling efforts, including unaccounted inter- and intraindividual variability in exposure patterns (Kromhout et al. 1993; Symanski et al. 2007) or from variability based on limited monitoring data. Once these different types and sources of measurement error are identified, bias analyses should be included to examine uncertainty due to the use of different exposure metrics in relation to what is known about the critical exposure period or evaluation of specific parameter estimates (e.g., half-life considerations of biological measures) or other modeling assumptions, such as the validity of the underlying input data (e.g., chemical monitoring data) and modeling data (e.g., fate and transport models) used to estimate exposure concentrations. Statistical techniques, both non-Bayesian and Bayesian, are available to allow for the correction of biased effect estimates resulting from exposure measurement error. Examples of non-Bayesian methods for accounting and adjusting for exposure measurement error include conditional likelihood methods (Guolo and Brazzale 2008; Lash and Fink 2003; Lash et al. 2009; Maldonado 2008; Stram et al. 2003) such as regression calibration (Spiegelman 2010) and conditional scores procedures (McShane et al. 2001), whereas Bayesian methods exist that can be used for both binary and continuous exposures (Espino-Hernandez et al. 2011; Liu et al. 2009; Prescott and Garthwaite 2005; Rice 2003).

*Develop improved methods for assessing exposures to multiple environmental chemicals and multiple routes of exposure.* Traditionally, risk assessments have focused primarily on single chemicals. However, this does not reflect human exposure conditions. There is a recognized need to focus on multimedia sources of exposure to individual chemicals as well as complex mixtures. This is an area of research where observational studies, such as epidemiology, are an improvement over experimental studies because they can more readily address multiple exposures simultaneously.

It is important that epidemiologists continue to develop and evaluate methods for assessing exposure to complex mixtures in order to better characterize exposure assessment and to allow for the evaluation of effect measure modification and confounding. This would establish a robust scientific database necessary to conduct cumulative risk assessments. The understanding of the relationships among complex exposures will require modeling of monitoring data and other exposure determinants and development of techniques for assessing exposures to mixtures that result in unbiased or minimally biased effect estimates (Carlin et al. 2013). In addition, approaches such as multivariate source receptor modeling represent promising avenues for assessing exposure to complex mixtures (Hopke 2010), although further work is needed to account for key sources of uncertainty in such models. The development of efficient, easily measured, cost-effective exposure surrogates for key mixtures of concern will be important, and will include its own challenges for identifying and quantifying exposure measurement error. For example, it will be important to understand how the type and structure of measurement error may differ across different individual mixture components or for a surrogate representing exposure to the whole mixture. Techniques are needed to characterize and adjust for exposure measurement error of chemical mixtures.

### Analytic Tools in Epidemiology

Given that epidemiologic data are key inputs for risk assessments, it is important to apply methodologies that better characterize validity and precision of study results. The methods considered can be broadly classified as *a*) frequentist methods to address study biases systematically and quantitatively; *b*) Bayesian statistical techniques, which utilize prior knowledge addressing causal hypotheses and estimation problems under evaluation; and *c*) computational methods (e.g., cross-validation, resampling techniques, and boosting and model ensemble techniques), which provide valid statistical inferences without requiring strong *a priori* modeling assumptions. Each of these broad approaches addresses validity and characterizes the uncertainty of results from a single study and extends to improved characterization of epidemiologic results in weight-of-evidence assessments.

The analytic methods group discussion included four specific areas that facilitate causal interpretation in epidemiology: *a*) the use of directed acyclic graphs [DAGs, diagrams consisting of variables connected by arrows or lines to depict often complex relationships (Joffe et al. 2012)]; *b*) summarizing epidemiologic results using Bayesian posterior distributions; *c*) strategies for quantitatively evaluating measurement error; and *d*) formally assessing causality as it relates to policy decisions. Each of these areas led to a set of recommendations. These areas served as the basis for further discussion of related topics such as primary versus secondary analyses, journal requirements, epidemiology curricula, and data sharing practices.

### Analytic Methods: Issues and Recommendations

*The application of DAGs should be encouraged more broadly.* Joffe et al. (2012) described how DAGs make explicit the assumed or estimated relationships among unobserved and measured variables, indicating the causal direction of the potential relationships. As described, DAGs are considered to be an appropriate method to illustrate causal hypotheses and to specify the structure of associations between variables of interest. They also provide a useful way to represent assumptions, especially conditional independence assumptions, necessary for statistical analyses and causal inference. Last, DAGs are helpful for determining which factors may be confounders or effect modifiers of an association between exposure and outcome (VanderWeele and Robins 2007). DAGs provide transparent representations of a hypothesis as well as justification for specific analytic strategies to be applied during the investigation, such as identification of causal intermediates. DAGs can also clarify methodologic challenges, such as illustrating selection bias (Flanders and Klein 2007; Hernán et al. 2004). We recommend that journal editors request that DAGs be included in supplemental material (Westreich and Greenland 2013).

*Incorporate prior knowledge through Bayesian methods.* Bayesian statistical analysis differs from frequentist methods in that Bayesian analyses use information that exists before study data are collected and analyzed (i.e., “prior” distributions) to update what can be learned about a specific problem after conducting a study by expressing the new state of knowledge as “posterior” distributions. Results from the literature or other data sources are used to specify the *a priori* distribution for any parameters, such as the size and direction of exposure–outcome associations and the extent of measurement error. Subsequently, the study results generated by the analysis provide an assessment of the conditional probability distribution of parameters of interest (the posterior distribution) by reconciling the data observed, the analytic model fitted to the study data, and the prior information incorporated into the analytic model (Bolstad 2007).

Bayesian techniques can also allow for simultaneous correction for sources of bias such as measurement error and confounding (de Vocht et al. 2009; Steenland and Greenland 2004) that are typically treated in isolation in current practice of epidemiology (Gustafson and McCandless 2010). Although these techniques are not routinely employed, specification of prior model probabilities by investigators is inherent in grant proposals, the introduction section of a study publication, and the subjective interpretation of results (Goodman 2001). Thus, it could be argued that current practice involves presenting Bayesian considerations of a research article, whereas the reported results often rely on frequentist analysis and qualitative interpretations (Pearce and Corbin 2013).

*Measurement error should not be ignored in any analysis of epidemiologic results and should be assessed using quantitative methods.* Measurement error is an almost universal limitation of epidemiologic studies and their analyses. The current practice of acknowledging it diffusely with a brief discussion that frequently invokes its theoretical impact, for example, that it is most likely to be nondifferential and results in potential for bias toward a null result, will not improve epidemiologic input into risk assessments. Strategies for quantitatively correcting for the bias resulting from measurement error are described in textbooks and can be readily implemented for many study designs. These include regression calibration, simulation–extrapolation, Bayesian approaches (Carroll et al. 1995; Gustafson 2004), and computational statistical approaches (e.g., multiple imputations, data augmentation, and expectation–maximization algorithms). Attention should be given to correcting for measurement error available in commonly used epidemiologic software platforms such as rcal in STATA (http://www.stata.com/merror/rcal.pdf) (Hardin et al. 2003) or *PROC CALIS* in SAS (SAS Institute Inc.). Peer reviewers and journal editors should expect formal quantitative assessments of measurement error and related biases, as well as correction for bias created by measurement error, rather than relying on qualitative discussions. In addition, adequate funding should be designated for exposure validation studies, and granting agencies should consider such validation studies as essential criteria for funding epidemiologic research (Heid et al. 2004).

*Distinguish associations from causes.* Formal causality assessments are important and influence policy decisions. The synthesis of epidemiologic studies can be the primary basis for regulation and policy actions. Without state-of-the-art analytic techniques being used more routinely in epidemiologic studies and other lines of evidence, the benefits and costs of recommended interventions or action could be misestimated and apparent cost-effective interventions may be ineffective. In particular, it is unwarranted to assume that a specific statistical association represents a causal effect, such that changing the predictor variable would result in a corresponding change in the outcome variable (Freedman 2004). Indeed, the distinction between structural and reduced-form equations in econometrics, and phenomena such as Simpson’s Paradox, demonstrate that (reduced-form) regression coefficients need not even have the same sign as corresponding causal coefficients, showing how a change in an explanatory variable would change the dependent variable (Pearl 2009).

Although epidemiologists are aware of basic threats to inferential validity from observational studies, there is little agreement, even among workshop participants, on whether epidemiologists should consider the policy implications of declaring an association to be causal. One view expressed by workshop participants was that epidemiologists should primarily conduct research that supports or refutes qualitative statements about causation, as in showing that an exposure “causes” a specific disease. This viewpoint emphasizes epidemiology’s role in hazard identification, that is, an early stage of risk assessment for which putative threats to health are identified as causal. Another viewpoint was that epidemiologic results could be used to conceptualize causation in the context of population health, for example, showing that some modifiable exposure is capable of causing important changes in health of population overall. This would more closely align epidemiology with the risk characterization phase of risk assessment in which costs and benefits of risk management interventions are weighted and risks are appraised quantitatively (Phillips 2001). Alternative outcomes analysis, an example of a technique that can provide important insights in distinguishing association from causation, could be more routinely applied in assessing causal inference and attributable risk estimation (Jager et al. 1990; Meijster et al. 2011a, 2011b; Thomsen et al. 2006). Alternative outcomes analysis allows for the conceptualization of causation in terms of causes of meaningful versus ignorable consequences, assuming these can be readily differentiated into one of these two options. Regardless of how epidemiologic data align with the risk assessment paradigm, epidemiologic practice should adopt methods that apply state-of-the-art techniques to address uncertainty and other study limitations and to help contextualize epidemiologic study results in terms of causality and public health intervention.

## Conclusions and Future Directions

Epidemiologic data are critical for risk assessment efforts but are rarely conducted with quantification of uncertainty, which may limit their use in risk assessments. The HESI Epidemiology Subcommittee workshop focused on strengthening the utility and application of epidemiology studies by recommending improvements in analytic methods, exposure assessment approaches, and other techniques to quantify and account for specific sources of uncertainty.

Several recommendations resulted from this effort. Specific statistical approaches and analytic techniques, such as increased use of quantitative bias analysis, DAGs, and Bayesian analyses, are available for improving the inferences drawn from epidemiologic results and are currently used infrequently. In addition, new methods may be needed for assessing exposure and characterizing uncertainty related to chemical mixtures. Other deliberations in the workshop highlighted the complete reporting of all data elements and analytic tables to permit others to conduct uncertainty analyses (either in supplemental material published by journals or through the investigators’ institution). Specifically, increased transparency of results would improve weight-of-evidence evaluations, and collaboration with researchers in other disciplines would improve study designs and analytic approaches, particularly for exposure assessment.

Although there are multiple strategies for quantifying and reducing measurement error, there are barriers for routinely applying these techniques. A key disincentive is that substantial time and effort can be required to conduct validation or reliability studies, which can put a strain on research budgets. There may also be a perception that analyses of exposure measurement error tends to decrease the estimated precision of reported results, thereby increasing the probability of a false-negative result (Blair et al. 2009). Blair et al. (2007) suggested that exposure measurement error and the resulting misclassification is more likely to be nondifferential by disease status in epidemiology studies and will most frequently result in false negatives through attenuation of effect estimates. This assumption is made despite evidence from statistical literature that the impact of exposure measurement error can be profound and complex and that it is difficult to anticipate its impact on effect estimates in an individual study (Gustafson 2004). Given that many manuscripts are routinely accepted without analyses quantifying uncertainty, validated exposure assessment, or use of advanced analytic methods, there is little incentive to adopt the recommendations made. Funding organizations, peer reviewers, and journal editors should be catalysts for change in this effort.

The discussions and recommendations from this workshop demonstrate that there are practical steps that the scientific community can adopt to strengthen epidemiologic data for decision making. Use of available methods to quantify and adjust for uncertainty will help reduce the potential impact of different sources of error and bias and help achieve better decisions for risk assessment, policy, and ultimately public health.
